# Genetics of stroke in a UK African ancestry case-control study: South London Ethnicity and Stroke Study

**DOI:** 10.1212/NXG.0000000000000142

**Published:** 2017-03-15

**Authors:** Matthew Traylor, Loes Rutten-Jacobs, Charles Curtis, Hamel Patel, Gerome Breen, Stephen Newhouse, Cathryn M. Lewis, Hugh S. Markus

**Affiliations:** From the Department of Medical and Molecular Genetics (M.T., C.M.L.), King's College London, Guy's Hospital; Stroke Research Group (M.T., L.R.-J., H.S.M.), Department of Clinical Neurosciences, University of Cambridge, Cambridge Biomedical Campus; and SGDP Centre (C.C., H.P., G.B., S.N., C.M.L.), Institute of Psychiatry, Psychology & Neuroscience, King's College London, UK.

## Abstract

**Objective:**

Despite epidemiologic data showing an increased stroke incidence in African ancestry populations, genetic studies in this group have so far been limited, and there has been little characterization of the genetic contribution to stroke liability in this population, particularly for stroke subtypes.

**Methods:**

We evaluated the evidence that genetic factors contribute to stroke and stroke subtypes in a population of 917 African and African Caribbean stroke cases and 868 matched controls from London, United Kingdom. We (1) estimated the heritability of stroke in this population using genomic-relatedness matrix-restricted maximum likelihood approaches, (2) assessed loci associated with stroke in Europeans in our population, and (3) evaluated the influence of genetic factors underlying cardiovascular risk factors on stroke using polygenic risk scoring.

**Results:**

Our results indicate a substantial genetic contribution to stroke risk in African ancestry populations (h^2^ = 0.35 [SE = 0.19], *p* = 0.043). Polygenic risk scores indicate that cardiovascular risk scores contribute to the genetic liability (odds ratio [OR] 1.09 [95% confidence interval (CI) 1.01–1.17], *p* = 0.029) and point to a strong influence of type 2 diabetes in large vessel stroke (OR 1.62 [95% CI 1.19–2.22], *p* = 0.0024). Single nucleotide polymorphisms associated with ischemic stroke in Europeans shared direction of effect in SLESS (*p* = 0.031), suggesting that disease mechanisms are shared across ancestries.

**Conclusions:**

Stroke in African ancestry populations is highly heritable and influenced by genetic determinants underlying cardiovascular risk factors. In addition, stroke loci identified in Europeans share direction of effect in African populations. Future genome-wide association studies must focus on incorporating African ancestry individuals.

Genome-wide association studies (GWAS) of cardiovascular diseases and their risk factors have greatly improved our understanding of their underlying causes. However, to date, studies in European ancestry populations have vastly eclipsed those in other populations. African and admixed African and European populations have been particularly neglected, despite the increased burden of cardiovascular diseases such as stroke,^1^ coronary heart disease (CHD), and type 2 diabetes (T2D) in these populations.^2,3^ Stroke, in particular, has higher incidence and mortality in African ancestry populations^4,5^; there is evidence that stroke incidence is decreasing in whites, but not in blacks.^6^ There is, therefore, considerable motivation for identifying the genetic component to stroke in African ancestry populations. One previous GWAS meta-analysis of 1,592 African American stroke cases from prospective cohorts totaling 14,746 individuals focused on the broad phenotypes of all stroke (AS), both ischemic and hemorrhagic, and of ischemic stroke (IS) alone.^7^ However, IS itself represents a syndrome which can be caused by many different pathophysiologic processes, with different genetic architectures reported for the 3 major IS subtypes: cardioembolic (CE), large vessel disease (LVD), and small vessel disease (SVD) stroke.^8^ As most stroke loci identified to date have been found to confer risk for specific stroke subtypes,^9,10^ characterizing the ancestry-specific genetic component of each of the stroke subtypes is of interest.

Barriers to performing GWAS in African ancestry populations until now have included the following: (1) the lack of suitable reference panels to adequately characterize and account for population differences in African populations and (2) the lack of suitable genotyping arrays that include appropriate single nucleotide polymorphisms (SNPs) for these populations. With the release of 1000 Genomes phase 3, which includes multiple African ancestry populations for the first time, and the development of cross-ancestry genotyping arrays, these barriers have now been removed.

We undertook genome-wide analyses of stroke and stroke subtypes in a population of black African and black African Caribbean individuals from London, United Kingdom, to identify genetic risk factors associated with stroke in this minority population. We estimated the heritability of stroke in this population and evaluated the evidence that SNPs associated with stroke in Europeans also influence risk in African ancestry individuals. In addition, we performed a genetic risk score (GRS) analysis to identify the contribution of genetic factors underling cardiovascular risk factors to the burden of stroke in this ethnic group.

## METHODS

### Cohort characteristics.

The cohort has been previously described in detail.^11^ Black patients with stroke were consecutively recruited from South London, United Kingdom. Three acute hospitals which cover a neighboring geographical region (Guy's and St Thomas' Hospitals, King's College Hospital, and St George's Hospital) were used to recruit patients between 1999 and 2010. All hospitals have a specialized stroke unit and a rapid-access TIA clinic. The ethnicity of participants was defined according to the 2001 definition derived from the UK Census. Participants were classified as black African or black African Caribbean.^12^

Recruitment of controls was by random selection from general practice lists in the catchment areas of St George's, Guys and St Thomas, and King's College Hospital NHS Trusts between 1999 and 2012. In addition, posters inviting healthy black African and black African Caribbean individuals were displayed in local leisure centers, general practice surgeries, churches, and community centers within a catchment area that included the London Borough of Wandsworth. Using community-based controls from the same catchment area as cases reduced the risk of selection bias, by ensuring that they were representative of the population producing cases. Inclusion criteria comprised self-reported “Black Caribbean” or “Black African” ethnicity and being free of clinical cerebrovascular disease.

In both cases and controls, risk factors were collected on a standard proforma, and blood pressure and body mass index were measured. A single consultant stroke neurologist (H.S.M.) derived Trial of Org 10172 in Acute Stroke Treatment (TOAST) subtypes for all patients.^13^ These were derived from data collected on the standard proforma as well as from review of all original brain imaging in all patients, combined with review of clinical notes, as appropriate. The presence of hypertension and diabetes was not used as a criterion in the diagnosis of TOAST subtypes to avoid any bias resulting from different risk factor prevalences. Patients with other determined causes of stroke, such as sickle cell disease, dissection, or vasculitis, were excluded from the study. Where patients had previous stroke, subtyping was performed on the current stroke. The presence of intracranial stenosis was determined by CT angiography or magnetic resonance angiography, where performed. Stenosis of greater than 50% in the arterial territory of the stroke was used as a criterion for determining intracranial stenosis as the pathophysiologic cause.

### Standard protocol approvals, registrations, and patient consents.

The study protocol had ethics approval (Wandsworth Local Research Ethics Committee), and informed consent was obtained from all participants.

### Genotyping and quality control.

Study participants from whom blood samples were obtained and who had consented to the study were included in the genetic analysis. DNA was extracted from blood in all samples. Data were genotyped at the Institute of Psychiatry, Psychology & Neuroscience BRC Genomics Facility on the Illumina Multi-Ethnic Genotyping Array (MEGA). SNPs were removed with excess missingness (>3%), deviation from Hardy-Weinberg equilibrium (*p* < 1e-6), or low minor allele frequency (<0.005). Individuals with excess missingness (>3%), low or high levels of heterozygosity, relatedness (pi-hat > 0.1875), and discordant phenotypic/genotypic sex information were removed. All quality control was performed using PLINK v1.90b2r. Principal components were calculated in combination with individuals from 1000 Genomes phase 3 populations using smartpca (EIGENSTRAT) on an LD-pruned subset of the data. Data were imputed to 1000 Genomes phase 3 reference set using SHAPEIT (v2.r778) (for phasing) and impute2 (v2.3.0) (for imputation). Prior to imputation, strand ambiguous SNPs (A/G and C/T) were removed.

### Heritability of stroke in an African ancestry population.

We used GREML (genomic-relatedness matrix-restricted maximum likelihood) approaches, implemented in the GCTA package,^14,15^ to evaluate the proportion of stroke trait variance (or SNP heritability) explained by genetic factors in SLESS. Briefly, the approach estimates the distant relatedness between all individuals in a data set and uses linear mixed models to estimate the proportion of trait variance explained by excess distant relatedness between cases compared to controls. As our sample size was limited, we restricted our analysis to the phenotype of AS vs controls. We included 10 ancestry-informative principal components to control for population structure and used the estimated population prevalence of stroke for England (2.3%) from a recent report.^16^ We removed individuals with distant relatedness above a threshold of 0.05.^14^ Analyses were performed using GCTA version 1.26.0.

### Cardiovascular risk factors GRS.

We identified SNPs associated with T2D, lipids, blood pressure, and CHD in populations of African or admixed African/European ancestry from recent publications.^17–21^ Thirty-one associations were identified (table e-1 at Neurology.org/ng); 7 for blood pressure, 2 for CHD, 16 for lipid levels (high-density lipoprotein [HDL], low-density lipoprotein, and triglycerides [TG]); and 6 for T2D. However, one SNP (chr11:116799496, associated with HDL) was rare and not well imputed in our data set. A second SNP, rs326, was associated with HDL and TG, so was only included once. In total, 29 SNPs were included in the risk score. We generated a risk score for each case and control in our population by summing the genotype dosage (between 0 and 2) of trait-increasing SNPs for all variants. The exception was for SNPs associated with HDL, for which we assumed trait-increasing SNPs would have a protective effect, and therefore coded them as such in the risk score. We tested association of the risk score with stroke using logistic regression, including 10 ancestry-informative principal components as covariates. Our primary analysis was for the phenotype of AS against controls—we used a *p* value of 0.05 to assess significance in this analysis. After assessing this, we performed analyses for each of the IS subtypes, and for intracerebral hemorrhage, and explored associations with the different traits that composed of the 31 SNP risk score. Analyses were performed using R Statistical Software (version 3.2.2).

### European stroke-associated SNPs in an African ancestry population.

We identified SNPs that have been associated at genome-wide significance with stroke and stroke subtypes from recent large-scale meta-analyses and evaluated them in SLESS in the subtype in which they were originally identified.^9,10,22–24^ Association analysis was performed on imputed genotype dosages using PLINK v1.90b2r, including age, sex, and the first 10 principal components as covariates.

## RESULTS

Cohort characteristics are presented in table 1. Across the SLESS study, 95.3% had a CT, 47.8% had an MRI, 98.6% had an ECG, 51.3% had an echocardiogram, and 96% had extracranial vessel imaging. Intracranial imaging was performed in 40.5% of patients, with intracranial stenosis found in 20.1% of patients.

**Table 1:**
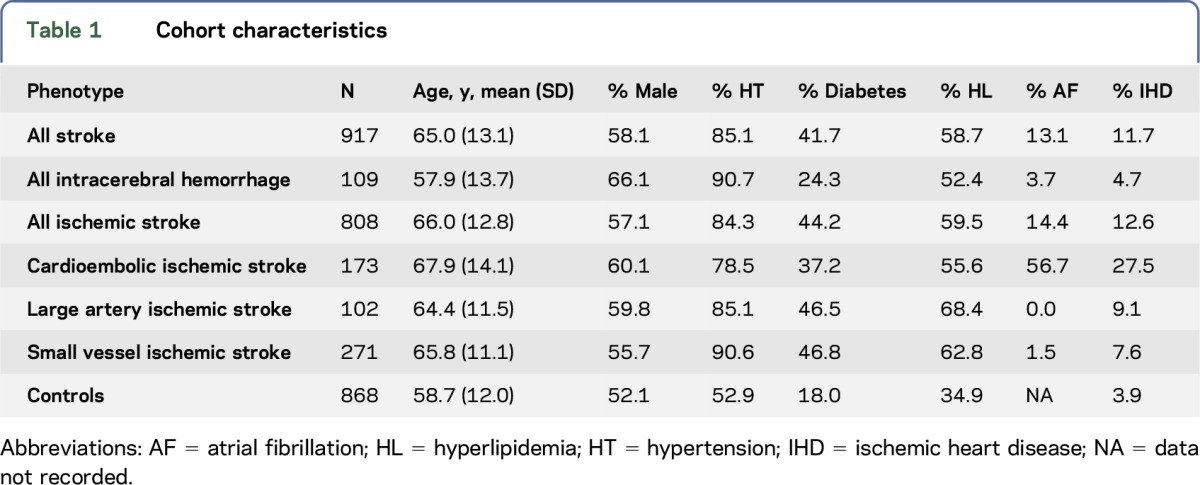
Cohort characteristics

One thousand eight hundred ninety-seven individuals were genotyped in total. After quality control procedures, there were 917 stroke cases and 868 controls, all of black African or black African Carribean ancestry. The majority of cases were black African Carribean (63.9%). The mean age in cases was 65.0 years (SD 13.1). The mean age was similar across ischemic subtypes but lower for intracerebral hemorrhage (57.9 years [SD 13.7]).

### Population structure.

We used principal component analysis, implemented in EIGENSTRAT to characterize the genetic ancestry of SLESS individuals, merging them with 1000 Genomes phase 3 samples for comparison, which includes African populations from Kenya, Nigeria, Sierra Leone, and Gambia; and African Caribbean populations from Barbados. There were differences in ancestry-informative principal components between SLESS black African and black African Caribbean individuals, with more European admixture in black African Caribbean individuals (PC1; *p* = 3.7 × 10^−18^) as might be expected given the demographic history of the Caribbean region. Principal component 6 broadly separated African populations by geographical location from East to West. We saw more segregation of SLESS black African samples with East African samples from 1000 Genomes. To ensure that population structure was adequately controlled for by including the first 10 principal components, we performed genome-wide association analyses in each phenotype, assessed the genomic inflation factor, and plotted QQ plots. In each analysis, inflation was well controlled (λ < 1.02), indicating that inflation due to population structure was well controlled (figures e-1–e-5).

### Heritability of stroke in an African ancestry population.

We next estimated the heritability of stroke in SLESS using GREML methods. With a relatedness threshold of 0.05 (equivalent to second-cousin relatedness), 161 individuals were removed (89 cases, 72 controls). We detected a genetic contribution to AS in SLESS (*p* = 0.043). Assuming a population prevalence of 2.3%, we estimate heritability to be 0.35 (SE 0.19). If the prevalence were assumed to be higher (4%), this estimate would rise to 0.41 (SE 0.23); whereas for a lower prevalence (1%), the estimate is 0.26 (SE 0.16). This compares to a heritability estimate of 0.18 in the largest analysis in Europeans to date.^8^

### Cardiovascular risk factor GRS.

**Figure 1:**
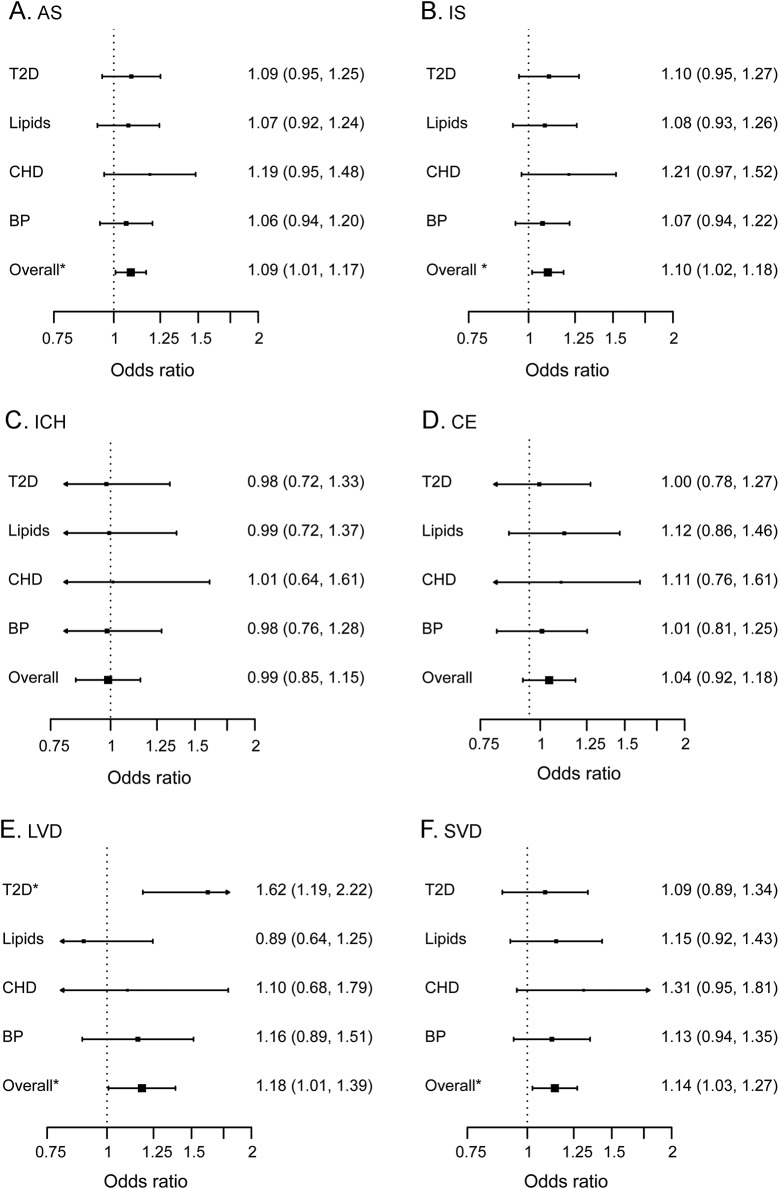
Association of genetic risk scores derived from cardiovascular risk factors with stroke phenotypes in the South London Ethnicity and Stroke Study (A) All stroke. (B) Ischemic stroke. (C) Intracerebral hemorrhage. (D) Cardioembolic. (E) Large vessel disease. (F) Small vessel disease. *p < 0.05; AS = all stroke; BP = blood pressure; CHD = coronary heart disease; ICH = intracerebral hemorrhage; IS = ischemic stroke; LVD = large vessel disease; Overall = all risk factors combined; SVD = small vessel disease; T2D = type 2 diabetes.

The GRS capturing SNPs associated at genome-wide significance in African ancestry populations with T2D, CHD, blood pressure, and lipid levels was associated with AS (odds ratio [OR] 1.09 [95% confidence interval (CI) 1.01–1.17], *p* = 0.029), with a slightly stronger association in IS (OR 1.10 [95% CI 1.02–1.18], *p* = 0.017, figure 1). Conversely, the risk score was not associated with intracerebral hemorrhage (OR 0.99 [95% CI 0.85–1.15], *p* = 0.89). We next explored the association of the risk score with IS subtypes. There was an association with SVD (OR 1.14 [95% CI 1.03–1.27], *p* = 0.016) and LVD (OR 1.18 [95% CI 1.01–1.39], *p* = 0.041) but not CE (OR 1.04 [95% CI 0.92–1.18], *p* = 0.51). In LVD, the association was driven by a strong association with T2D SNPs (OR 1.62 [95% CI 1.19–2.22], *p* = 0.0024).

### European stroke-associated SNPs in an African ancestry population.

All 6 associations with IS or IS subtypes in Europeans shared the same direction of effect in SLESS (*p* = 0.031 from binomial test). However, none of the SNPs associated with stroke or stroke subtypes in Europeans were associated with stroke in SLESS (table 2). For most of the SNPs, allele frequencies were similar in European populations and in SLESS. A notable exception was rs10744777 (12q24.12), which was much rarer in SLESS (frequency of T allele = 0.05) than in Europeans (frequency of T allele = 0.68). Frequencies of all SNPs were comparable to those from matched reference populations.^25^ One SNP, rs4471613, was reported to be associated with stroke in an African American population at genome-wide significance, albeit without replication.^7^ We found no evidence that this locus was associated with stroke in SLESS (OR 1.05 [95% CI 0.62–1.77]; *p* = 0.85).

**Table 2:**
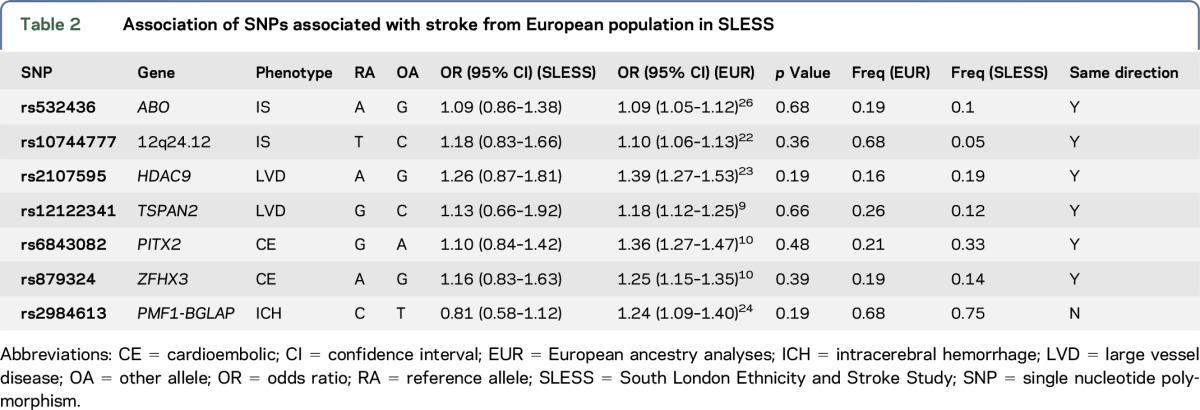
Association of SNPs associated with stroke from European population in SLESS

## DISCUSSION

We performed genome-wide analyses to characterize the genetic contribution to stroke in black African and black African Caribbean individuals from the local population in London, United Kingdom. Our results provide novel insights into the genetic contribution to stroke in this population. First, they indicate that genetic factors are a contributor to stroke liability in this ethnic group. Our estimate of SNP heritability was greater than that observed in the largest such analysis in Europeans,^8^ which might indicate a stronger influence of genetics in this population. However, our estimate lacked precision, reflecting the small sample sizes; therefore, a much larger sample would be required to determine this increase conclusively. Second, our results show that the genetic component to cardiovascular risk factors such as T2D, CHD, lipid levels, and hypertension contributes to stroke risk in this population. This mirrors findings in European ancestry populations^26,27^ and therefore indicates similarities in underlying risk factors across populations. The risk score was most strongly associated with the LVD and SVD subtypes of stroke—and in LVD, this was driven by a strong association with T2D, for which there is an increased burden in this population.^28^ Our results might, therefore, suggest a particularly strong influence of T2D on LVD in this population. In addition, the direction of effect of the association of a lipid GRS with LVD was in the opposite direction to what might be expected. Given the small number of LVD cases in our analysis (N = 109), further replication of these findings is warranted. Third, we investigated SNPs previously reported as being associated with stroke, mostly from European-only analyses, in SLESS. Although no individual SNP was significant, all IS associations were in the same direction of effect, suggesting that disease mechanisms are likely to be shared across ancestries.

This study has limitations. Our sample size (917 cases and 868 controls) was relatively limited for GWAS, meaning that we were unable to calculate heritability of stroke in disease subtypes. The low sample size for these analyses meant that the GCTA algorithm did not converge. Larger studies will be required to perform these analyses and to formally compare estimates of heritability between different ancestral populations. Our GRS were based on SNPs identified in studies to date in African ancestry populations. As studies in African ancestry populations grow larger, more SNPs will be identified, and this will no doubt influence the results from an equivalent analysis. In all analyses, we grouped black African and black African Caribbean individuals together to increase power. Risk factor profiles differ in these 2 populations, which might modulate the influence of genetics on risk of stroke in the 2 groups. We were underpowered to assess these differences. However, the GRS results for AS were not significantly different in the African Caribbean subgroup to the overall results (OR 1.05 [95% CI 0.97–1.14] compared to OR 1.09 [95% CI 1.01–1.17]), which might point to similarities between the groups. A single individual performed AS subtyping. This is a common approach in genetic studies of stroke; although intraobserver agreement has been shown to be relatively high, there remains some potential for consistent misclassification.

As loci identified in GWAS increase in number, the priority in complex disease research will move to interpreting associated loci and identifying the causal variants. African ancestry populations will perform a vital role in this exercise as, due to the lower linkage disequilibrium in these populations, fine mapping techniques can be more successfully used,^29^ which will help to identify which specific variants are associated with disease. This population, and the results obtained from these analyses, will help to guide such future multiethnic genetic studies in stroke.

## Supplementary Material

Data Supplement

## GLOSSARY

AS: all stroke
CE: cardioembolic
CHD: coronary heart disease
CI: confidence interval
GREML: genomic-relatedness matrix-restricted maximum likelihood
GRS: genetic risk score
GWAS: genome-wide association studies
HDL: high-density lipoprotein
IS: ischemic stroke
LVD: large vessel disease
OR: odds ratio
SLESS: South London Ethnicity and Stroke Study
SNP: single nucleotide polymorphism
SVD: small vessel disease
T2D: type 2 diabetes
TG: triglyceride
