# Supplementary material for: Genetics of stroke in a UK African ancestry case-control study: South London Ethnicity and Stroke Study
Source: Neurol Genet. 2017 Mar 15;3(2):e142. doi: 10.1212/NXG.0000000000000142 (PMC5354108; doi:10.1212/NXG.0000000000000142)
Supplement: Data Supplement [file supp_3_2_e142__index.html]

Data Supplement 

# Genetics of stroke in a UK African ancestry case-control study

## Data Supplement

**Files in this Data Supplement:**

- Figures\_e-1-e-5.docx
- Table e-1
