# Supplementary material for: Genetics of stroke in a UK African ancestry case-control study: South London Ethnicity and Stroke Study
Source: Neurol Genet. 2017 Mar 15;3(2):e142. doi: 10.1212/NXG.0000000000000142 (PMC5354108; doi:10.1212/NXG.0000000000000142)

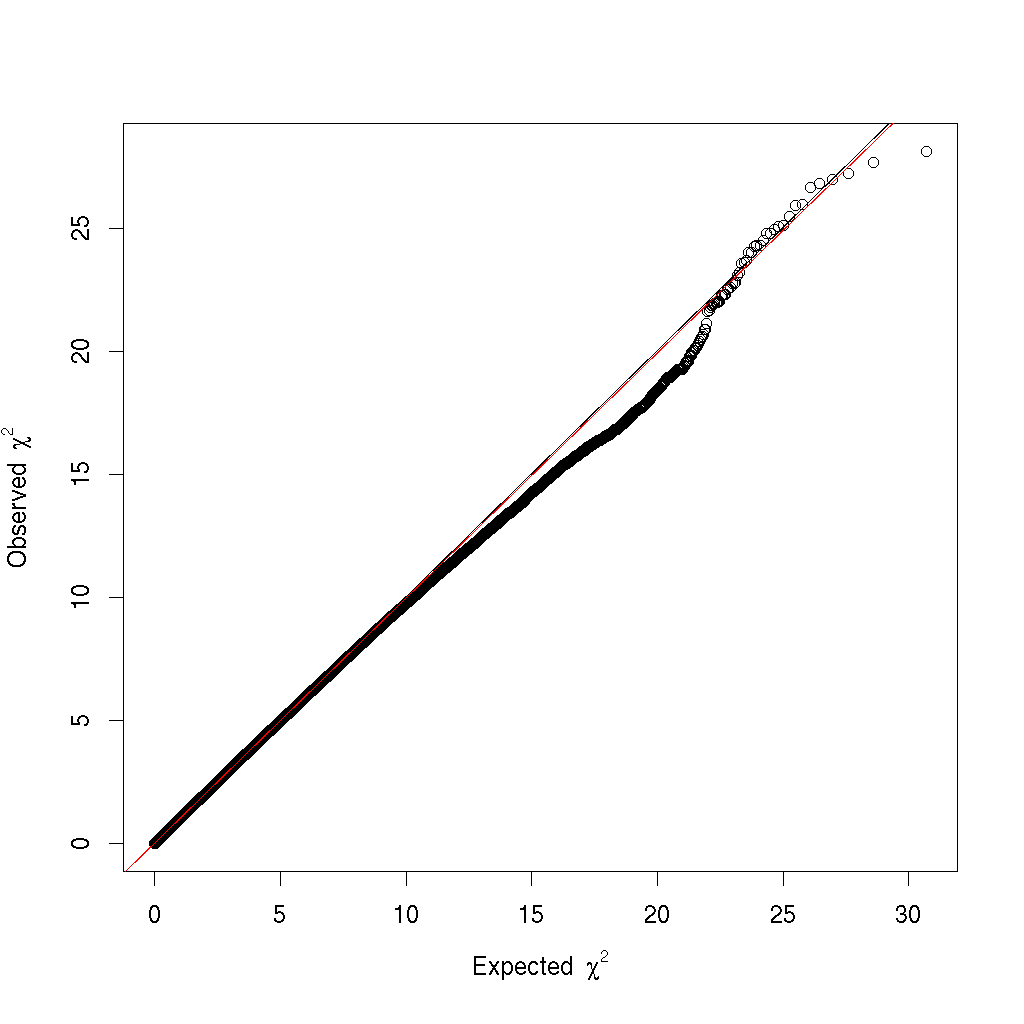
Figure e-1 – QQ-plots of Observed against Expected association statistics for all ischaemic stroke

Figure e-2 – QQ-plots of Observed against Expected association statistics for all stroke


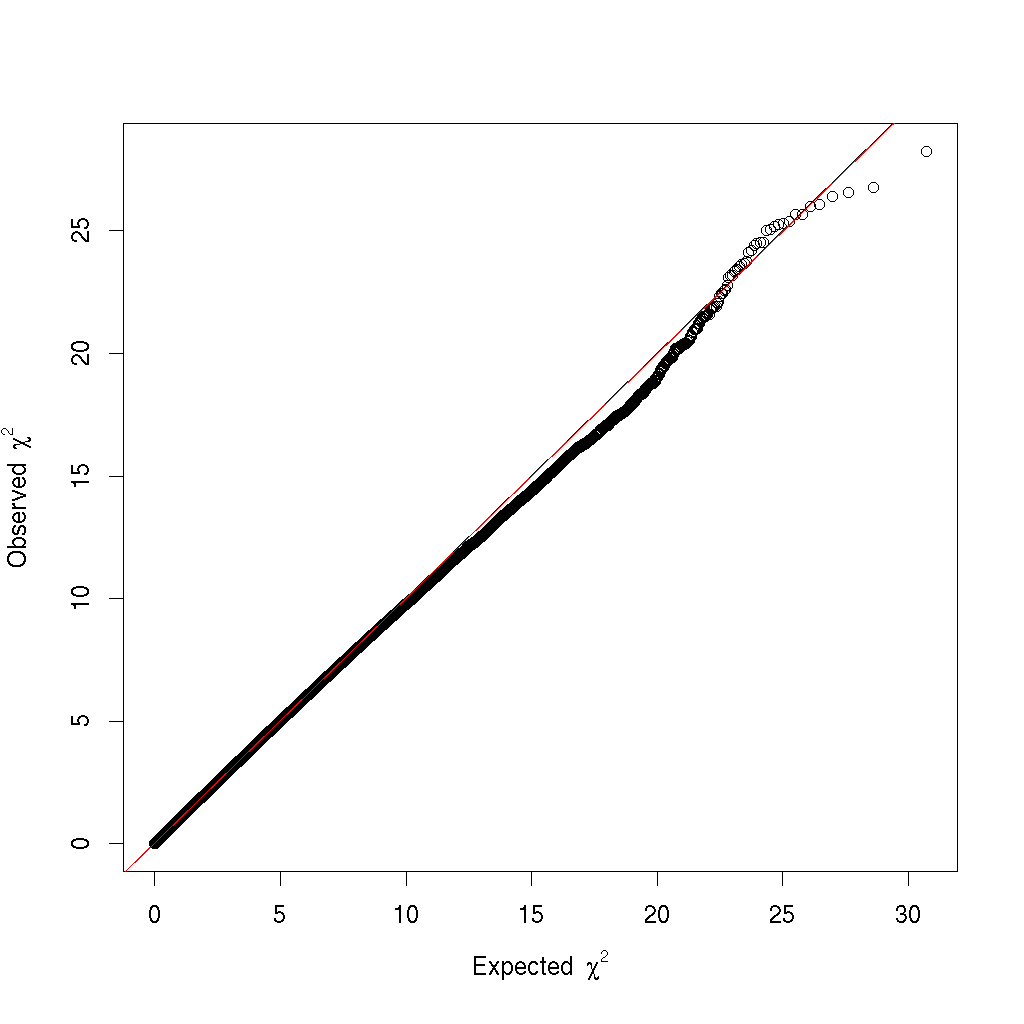


Figure e-3 – QQ-plots of Observed against Expected association statistics for cardioembolic stroke


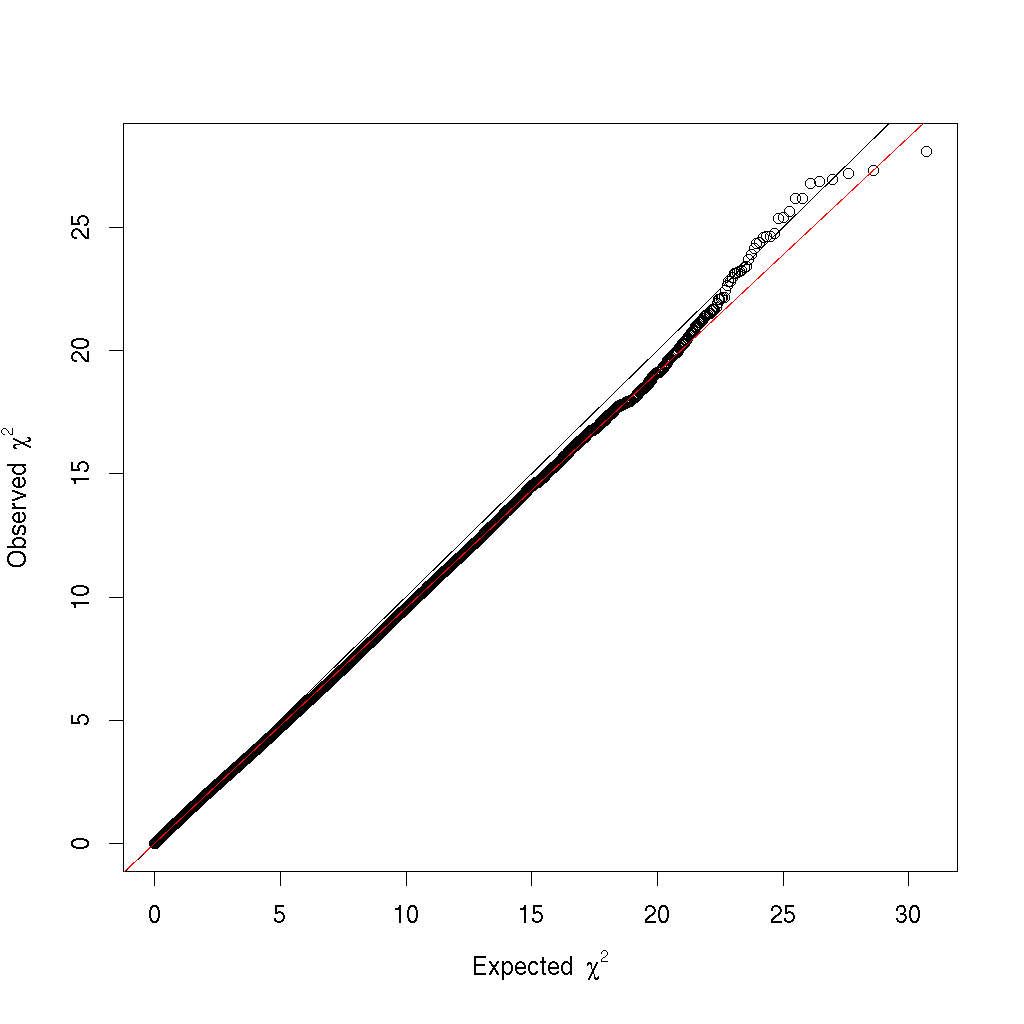


Figure e-4 – QQ-plots of Observed against Expected association statistics for large vessel stroke


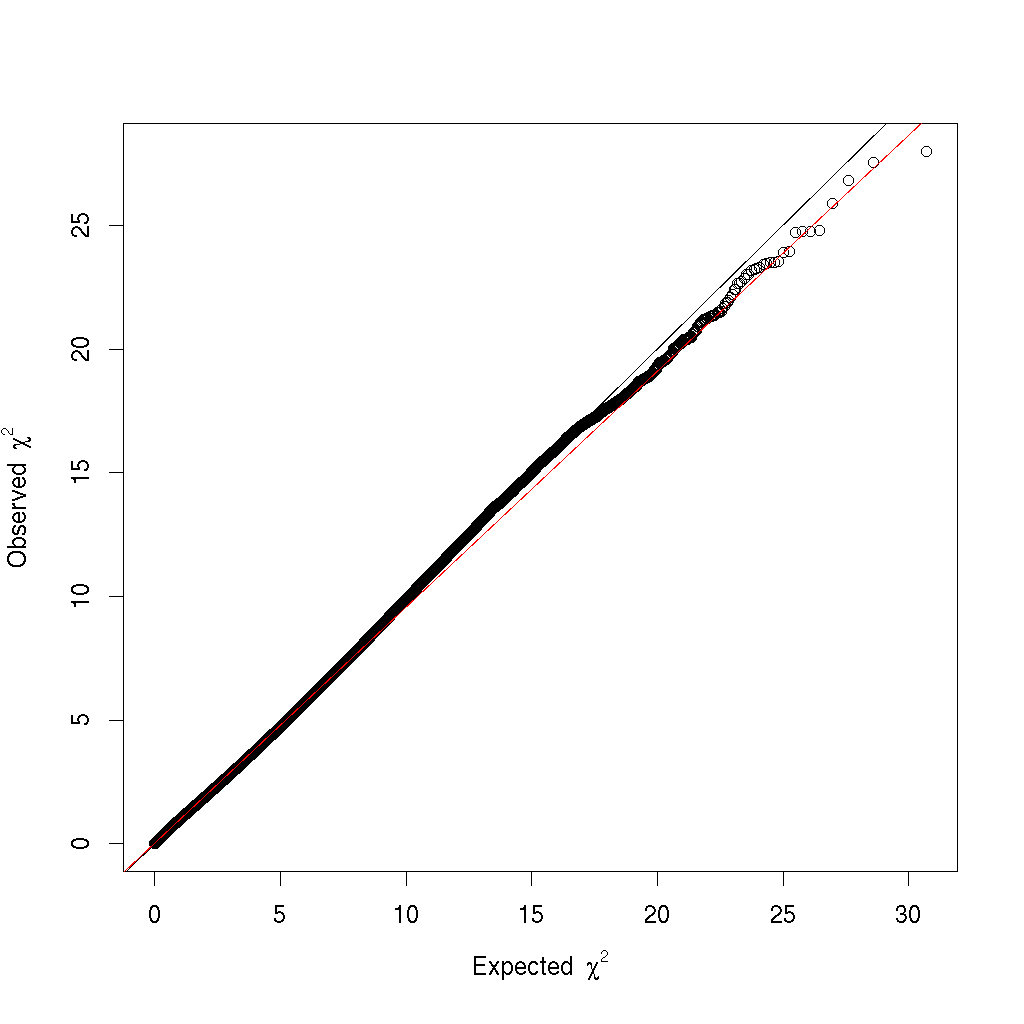


Figure e-5 – QQ-plots of Observed against Expected association statistics for small vessel stroke


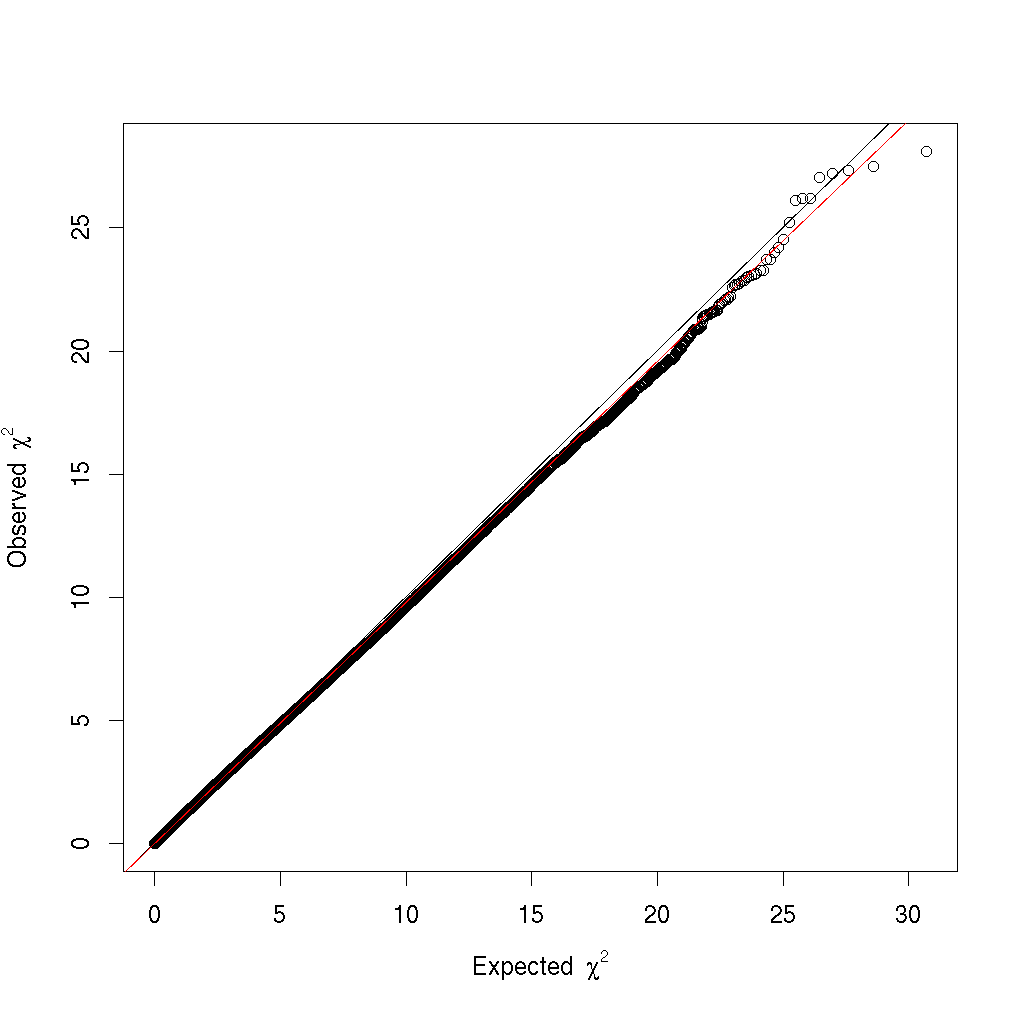

Supplement: Data Supplement [file supp_3.2.e142_Figures_e-1-e-5.docx]
